# A Case of Statin-Induced Rhabdomyolysis After Seven Years of Statin Therapy

**DOI:** 10.7759/cureus.102027

**Published:** 2026-01-21

**Authors:** Claudia R Silver, Bryce Somer

**Affiliations:** 1 Medical School, Florida State University College of Medicine, Sarasota, USA; 2 Internal Medicine, Florida State University College of Medicine, Sarasota, USA

**Keywords:** acute kidney injury, myopathy, rhabdomyolysis, statin, statin-induced myopathy

## Abstract

Rhabdomyolysis is a well-known but uncommon condition that can result from various causes, including overexertion, trauma, immobilization, toxins, and certain drugs. Release of muscle cell contents, including creatine kinase (CK) and myoglobin, can lead to fatal complications, including disseminated intravascular coagulation (DIC), arrhythmias, and renal failure. The most well-known cause of rhabdomyolysis is trauma, yet many patients are aware of the association between statins and rhabdomyolysis. Typically, cases of statin-induced rhabdomyolysis occur within a month of starting the medication. Here, we report the case of a 77-year-old woman who presented with myalgias and weakness and was subsequently found to have a CK of 23,203 U/L, transaminitis, and acute kidney injury, indicative of rhabdomyolysis that developed after seven years of previously well-tolerated continuous statin therapy.

## Introduction

Rhabdomyolysis is a condition that refers to the damage and resultant breakdown of muscle tissue, resulting in the release of muscle cell contents such as creatine kinase (CK), myoglobin, and potassium into the bloodstream and urine. It most commonly presents with myalgias, weakness, and dark urine. Life-threatening complications can include acute renal failure, electrolyte imbalances leading to arrhythmias, and, in severe cases, disseminated intravascular coagulation (DIC) [1]. Common triggers include strenuous exercise, crush injuries, infection, seizures, prolonged immobilization, toxins, and medications. An estimated 26,000 cases of rhabdomyolysis are reported annually, with roughly 85% of cases attributable to traumatic injury [2,3]. While statins are a known risk factor for the development of rhabdomyolysis, the reported risk is <0.1% [4]. In patients who do go on to develop rhabdomyolysis, the average onset timing following the initiation of treatment is generally less than a month, with rosuvastatin yielding a result of 30 days [5]. Here, we report a rare case of delayed-onset statin-induced rhabdomyolysis, illustrating the potential for rhabdomyolysis even after long-term statin therapy and emphasizing the need for ongoing clinical vigilance. 

## Case presentation

A 77-year-old Caucasian woman presented to our outpatient Internal Medicine clinic for one week of generalized weakness and myalgias in her lower extremities and back. The weakness began in her legs, and she began to have difficulty crossing her legs and getting into her car. She endorsed new-onset shortness of breath associated with activity and dizziness. She denied chest pain. She stopped taking her losartan for a couple of nights and did not notice any improvement. She denied recent strenuous exercise, trauma, infection, dehydration, or new medications. Past medical history is significant for chronic kidney disease (CKD) stage 3, coronary artery disease (CAD), hypertension, melanoma of the right lower extremity, intervertebral disc degeneration, nephrolithiasis, and hypercholesterolemia. Medications include rosuvastatin for the past seven years (dosage of 40 mg nightly for the past three years), losartan 25 mg daily, and 81 mg aspirin.

At the time of presentation, her vitals were as follows: temperature 97.2 degrees Fahrenheit, oxygen saturation of 96% on room air, blood pressure 102/62 mmHg, and heart rate 72 beats/minute. Furthermore, her body mass index (BMI) was 21.72 kg/m². Orthostatic vital signs were negative. On physical exam, she appeared fatigued and in discomfort. Global strength was mildly decreased at 4/5, but deep tendon reflexes were intact. There was point tenderness of the upper lumbar and lower thoracic spine, but straight leg raise was normal. Labs including C-reactive protein (CRP), CK, erythrocyte sedimentation rate (ESR), and a renal profile were ordered to rule out myopathies. 

Labs came back with the following: CK 23,203 U/L, CRP <0.5 mg/dL, ESR 33 mm/hr, blood urea nitrogen (BUN) 35 mg/dL, creatinine 2.02 mg/dL, and potassium 2.9 mmol/L. Transaminases were elevated as well, with aspartate aminotransferase (AST) of 582 U/L and alanine aminotransferase (ALT) of 379 U/L consistent with muscle injury. The patient was contacted and directly admitted to the hospital. Urinalysis findings were suggestive of myoglobinuria, with 3+ blood on dipstick and 0-3 red blood cells per high-power field. She was treated with rest and aggressive fluid resuscitation. Her rosuvastatin was discontinued, and losartan was held for renal safety. She saw significant improvement in her symptoms as well as laboratory markers, with her CK improving from 27,111 U/L at its peak on hospitalization day 1 to 5,186 U/L on the day of discharge, day 3. Her renal function improved as well, with creatinine returning to 1.56 mg/dL at the time of discharge near her baseline of 1.40 mg/dL. No alternative etiology for her rhabdomyolysis was found, and it was presumed to be statin-related. The patient was advised to avoid statin therapy indefinitely.

The patient continued to see improvement in her symptoms after discharge and has remained off of statin therapy. Follow-up labs one week after her hospitalization showed continued improvement in her CK at 358 U/L, CRP <0.5 mg/dL, and ESR 30 mm/hr. Renal function further improved with creatinine of 1.23 mg/dL, and aminotransferases improved as well with AST of 42 U/L and ALT of 79.2 U/L. Months after her hospitalization, her CK had normalized at 118 U/L. Her creatinine had improved to 1.03 mg/dL, and her AST and ALT had normalized as well, to 27 and 16 U/L, respectively. Additional antibody testing was completed to rule out immune-mediated myopathies including antinuclear antibody (ANA), anti-Ro (SSA) antibody, anti-La (SSB) antibody, anti-Jo antibody, and 3-hydroxy-3-methylglutaryl-coenzyme A reductase (HMGCR) antibody all of which came back within normal limits. The patient has not had any recurrence of symptoms. Laboratory values are summarized in Table 1. 

**Table 1:** Summary of laboratory values during hospitalization and follow-up "-" indicates laboratory value not obtained. CK: creatine kinase; CRP: C-reactive protein; ESR: erythrocyte sedimentation rate; BUN: blood urea nitrogen; AST: aspartate aminotransferase; ALT: alanine aminotransferase

Laboratory parameter	Reference range	Initial presentation (day 0)	During hospitalization (day 1)	Day of discharge (day 3)	1-week follow-up	2-month follow-up
CK (U/L)	30-223	23,203	27,111	5,186	358	118
CRP (mg/dL)	<0.5	<0.5	<0.5	<0.5	<0.5	<0.5
ESR (mm/hr)	0-30	33	33	30	30	-
BUN (mg/dL)	7-25	35	36	15	21	22
Creatinine (mg/dL)	0.6-1.3	2.02	2.02	1.56	1.23	1.03
Potassium (mmol/L)	3.5-5.1	2.9	2.9	4.0	4.5	4.2
AST (U/L)	10-40	582	582	-	42	27
ALT (U/L)	7-56	379	379	-	79	16

## Discussion

Statins are first-line therapy for hypercholesterolemia due to their efficacy in reducing cholesterol and preventing cardiovascular disease in conjunction with their favorable safety profile. Their need continues to grow alongside the incidence of dyslipidemia, and they are now the most widely prescribed drug class worldwide [6]. A rare but commonly known and feared side effect is rhabdomyolysis, with most cases being discovered through case reports rather than clinical trials. As previously mentioned, the reported risk for statin-induced rhabdomyolysis is <0.1% [4]. A retrospective cohort study looking at over 252,460 statin users found only 24 patients total who had been hospitalized for rhabdomyolysis [7]. Most cases of statin-induced rhabdomyolysis are seen when statins are prescribed in combination with other drugs such as fibrates that potentiate their effects on muscle [8]. When statins alone do lead to rhabdomyolysis, effects are usually seen within a month or less of their initiation [5]. However, as seen in our case, there is potential for statin-induced rhabdomyolysis even after long-term usage. 

While the primary mechanism of statin-induced rhabdomyolysis is not entirely understood, statins are believed to potentiate skeletal muscle necrosis through a decrease in ubiquinone (coenzyme Q). The HMG-CoA pathway produces cholesterol and ubiquinone, among other essential molecules. Ubiquinone is a component of the mitochondrial electron transport chain, facilitating the transfer of electrons and therefore playing a crucial role in cellular energy production. Statins inhibit the HMG-CoA pathway and subsequently may inhibit ubiquinone and consequently cellular energy production, leading to mitochondrial dysfunction and muscle cell death [9]. This can manifest as weakness and myalgias, among many other symptoms. 

The presentation of rhabdomyolysis can be vague, especially in the absence of a known external cause such as crush injuries or excessive exercise. Most commonly, patients will present with muscle weakness, myalgias, lower extremity edema, hyperkalemia, and potentially darkened urine secondary to myoglobinuria [10]. It should be noted that, while electrolyte disturbances are common, the hypokalemia seen in our case is an atypical laboratory finding. The diagnosis is confirmed with a CK ≥5 times the upper limit of normal (often >1000 U/L). Other important measures include serum myoglobin, urinalysis to assess for myoglobinuria, and a full metabolic panel. Commonly reported risk factors include male gender, African-American race, those <10 years or >60 years of age, and those with a BMI >40 kg/m². In this case, age was the sole risk factor. If caught and worked up promptly, the prognosis is excellent. Treatment consists of supportive care including aggressive fluid replacement using crystalloid solution to prevent or treat acute kidney injury, immediate cessation of the offending agent, avoidance of nephrotoxic or renally cleared drugs, and electrolyte repletion as needed, as was performed in this case allowing for a full recovery [11]. 

## Conclusions

Our case highlights a rare case of suspected delayed-onset statin-associated rhabdomyolysis. The presentation of myalgias and weakness is non-specific and allows for a broad differential. Rhabdomyolysis should be considered in any individual with progressive myalgias and weakness, as early detection and treatment allow for a favorable prognosis. Treatment includes aggressive fluid resuscitation, supportive care for acute kidney injury, electrolyte monitoring and correction, discontinuation of medications associated with rhabdomyolysis, and individualized lipid-lowering alternatives.
